# A 30+ year AVHRR Land Surface Reflectance Climate Data Record and its application to wheat yield monitoring

**DOI:** 10.3390/rs9030296

**Published:** 2017-03-21

**Authors:** Belen Franch, Eric F. Vermote, Jean-Claude Roger, Emilie Murphy, Inbal Becker-Reshef, Chris Justice, Martin Claverie, Jyoteshwar Nagol, Ivan Csiszar, Dave Meyer, Frederic Baret, Edward Masuoka, Robert Wolfe, Sadashiva Devadiga

**Affiliations:** 1 Department of Geographical Sciences, University of Maryland, College Park MD 20742, United States; 2 NASA Goddard Space Flight Center, 8800 Greenbelt Road, Greenbelt, MD 20771, United States; 3 NOAA Center for Satellite Applications and Research, College Park, MD 20746, USA; 4 USGS EROS Data Center, Sioux Falls, SD, 57198, USA; 5 INRA, Unité Environnement Méditerranéen et Modélisation des Agro-Hydrosystèmes (UMR1114), Domaine St Paul, Site Agroparc, 84914 Avignon Cedex 09, France; 6 Science Systems and Applications Inc. Maryland, USA

**Keywords:** AVHRR, LCDR, MODIS, surface reflectance, yield monitoring

## Abstract

The Advanced Very High Resolution Radiometer (AVHRR) sensor provides a unique global remote sensing dataset that ranges from the 1980’s to the present. Over the years, several efforts have been made on the calibration of the different instruments to establish a consistent land surface reflectance time-series and to augment the AVHRR data record with data from other sensors such as the Moderate Resolution Imaging Spectroradiometer (MODIS). In this paper, we present a summary of all the corrections applied to the AVHRR Surface Reflectance and NDVI Version 4 Product, developed in the framework of the National Oceanic and Atmospheric Administration (NOAA) Climate Data Record (CDR) program. These corrections result from assessment of the geo-location, improvement of the cloud masking and calibration monitoring. Additionally, we evaluate the performance of the surface reflectance over the AERONET sites by a cross-comparison with MODIS, which is an already validated product, and evaluation of a downstream Leaf Area Index (LAI) product. We demonstrate the utility of this long time-series by estimating the winter wheat yield over the USA. The methods developed by [1] and [2] are applied to both the MODIS and AVHRR data. Comparison of the results from both sensors during the MODIS-era shows the consistency of the dataset with similar errors of 10%. When applying the methods to AVHRR historical data from the 1980’s, the results have errors equivalent to those derived from MODIS.

## 1. Introduction

The surface reflectance product is a critical input for generating downstream products such as Vegetation Indices (VI), Leaf Area Index (LAI), Fraction of Absorbed Photosynthetically Active Radiation (FAPAR), Bidirectional Reflectance Distribution Function (BRDF), Albedo, and Land Cover. A surface reflectance Land Climate Data Record (LCDR) needs to be of the highest possible quality, so that minimal uncertainties propagate in the dependent/downstream products. The generation of such a record necessitates the use of multi-instrument/multi-sensor science-quality data record and a strong emphasis on data consistency, which in this study is achieved by careful characterization and processing of the original data, rather than degrading and smoothing the dataset. As a consequence, the LCDR needs to be derived from accurately calibrated top of the atmosphere reflectance values that are precisely geo-located, carefully screened for clouds and cloud shadows, corrected for atmospheric effects using a radiative transfer model-based approach and finally corrected for directional effects. All these steps are necessary, as spurious trends will appear in the data record if the above effects are not corrected for.

The first requirement for accurate atmospheric correction is a proper absolute calibration of the instrument. Calibration errors propagate through the whole atmospheric correction chain, in particular through the aerosol inversion and impact most of the bands in the visible part of the spectrum and subsequent downstream products. It is very important therefore to assess instrument performance and independently monitor calibration. The Advanced Very High Resolution Radiometer (AVHRR) remains an important data source for the study of long-term variations in land surface properties as it provides the longest time-series of global satellite measurements [3]. [4] presented a method for absolute calibration of the red and near-infrared channels of AVHRR. It was based on a combination of observations over remote ocean areas and over highly reflective clouds located in the tropics over the Pacific Ocean. Later, [5] validated these results using a stable Saharan desert site and data from MODIS. The agreement between MODIS and AVHRR was better than 1%. Inter-comparison of the MODIS Aqua and AVHRR for the 2000–2014 period reported in this paper has further enabled refinement of the AVHRR record. Using state-of-the-art algorithms for geo-location, calibration, cloud screening, atmospheric and surface directional effect correction we have been able to achieve the most consistent data record possible. Such a long data record allows for the development of several applications involving evaluation of trends in surface properties (e.g. [6–8]). During the last several years, agricultural monitoring using remote sensing data has gained increasing interest among the science community mainly since the development in 2011 of the Group on Earth Observations Global Agricultural Monitoring (GEOGLAM) initiative. The main objective of GEOGLAM is to strengthen global agricultural monitoring by improving the use of remote sensing tools for crop production projections and weather forecasting. In this context we demonstrate the performance of the LCDR, by applying the yield model described in [1] and [2] to the V4 series of the AVHRR data records.

In this paper, we present the latest improvements of the AVHRR BRDF corrected surface reflectance and NDVI Version 4 products by assessing the accuracy of geolocation (section 3.1), calibration (section 3.2), cloud mask (section 3.3) and the final surface reflectance product using AERONET data (section 3.4) and cross-comparing it to MODIS Aqua (section 3.5). Additionally, we evaluate the performance of the LAI, which is derived using surface reflectance (section 3.6). Finally, an application of the product to estimate the winter wheat yield in the USA from the 1980’s is presented in section 3.7.

## 2. Materials

### 2.1. Land Climate Data Record (LCDR)

This work builds on previous efforts by [9] that created the first three versions of the consistent long-term land data records spanning a time period of 1981 – 2000 through processing and reprocessing of the AVHRR Global Area Coverage (GAC) data. The NASA LCDR detailed in [9] contains gridded daily surface reflectance and brightness temperatures derived from processing of the data acquired by the AVHRR sensors onboard four NOAA polar orbiting satellites: NOAA-7, −9, −11 and −14. Daily surface reflectance from the AVHRR channels 1 and 2 (at 640 and 860 nm) is a NOAA Climate Data Record (CDR). These data records are produced in a geographic projection at a spatial resolution of 0.05 degree similar to the Climate Modelling Grid (CMG) used in processing of the daily MODIS Surface Reflectance CMG data MOD09CMG/MYD09CMG.

With substantial improvements, the Version 4 Land Surface CDR products were produced by the NASA Goddard Space Flight Center (GSFC) and the University of Maryland. The Version 4 series extended the time period of the records to the present day through processing of the AVHRR data from the NOAA-16, 17, 18 and 19 with additional improvements to the Version 3. Improvements include better geolocation accuracy achieved by using One-Line-Element (OLE) instead of Two-Line-Element (TLE) for ephemeris, use of center of each grid as the reference to be consistent with other heritage records such as from MODIS on-board the Earth Observing System (EOS) satellites, and use of a weighted average of available observations instead of the one best sample used in Version 3. Version 4 was produced by reprocessing the raw GAC dataset for each instrument.

### 2.2. MODIS daily climate model grid (CMG) time-series

This study uses the MODIS CMG daily surface reflectance Collection 6 data (M{OY}D09CMG) distributed by the Land Processes Distributed Active Archive Center (LP DAAC, https://lpdaac.usgs.gov/products/modis_products_table), which are gridded in the linear latitude, longitude projection at 0.05° resolution (5600 m at the Equator). Science Data Sets (SDSs) provided for this product include surface reflectance values for bands 1–7, brightness temperatures for bands 20, 21, 31, and 32, solar and view zenith angles, relative azimuth angle, ozone, granule time, quality assessment, cloud mask, aerosol optical thickness at 550 nm and water vapor content.

### 2.3. Methods

#### 2.3.1. Geolocation

The purpose of geolocation assessment is to identify any errors by comparing the images to control points that can be easily traceable. Thus, in order to assess the accuracy of the geolocation of a given sensor, we used ‘coastal chips’ as a reference, which were selected manually using the MODIS CMG product. This approach has been proven very useful for the AVHRR dataset, where the error could be significant and the drift of the clock onboard the NOAA satellites leads to a desynchronization between the satellite clock and the tracking station clock [13].

#### 2.3.2. Calibration monitoring

The approach relies on using the multi-year MODIS Terra data set to derive spectral and directional characterizations of stable desert sites that can be used as invariant targets. A candidate list of such targets is provided in [15]. Subsets of MODIS Terra data are collected and undergo a rigorous screening based on the quality flags (cloud, cloud shadow, adjacent cloud, high aerosol or snow). The directional characterization is derived using the MODIS Bidirectional Reflectance Distribution Function (BRDF) algorithm that relies on a kernel-driven linear BRDF model, defined as a weighted sum of three kernels representing basic scattering types: isotropic scattering, radiative transfer-type volumetric scattering based on the Ross-Thick function and geometric-optical surface scattering based on the Li-Sparse model [16]. Using the site directional characterization, we compute a surface reflectance at the needed acquisition time and viewing conditions. Using the data corrected for directional effect we are also able to spectrally characterize the sites at the MODIS central wavelengths and account for spectral difference between MODIS and the AVHRR given the relatively broad AVHRR bands only for each particular site. Atmospheric parameters (surface pressure, gaseous content, water vapor, aerosol optical thickness) obtained from assimilated data, MODIS data, MODIS-like and/or ground measurements are then used in conjunction with the 6S radiative transfer code [17] to determine the target sensor (MODIS-Aqua, AVHRR) Top Of Atmosphere (TOA) reflectance. The computed reflectance is compared to the acquired reflectance to infer changes in the instrument calibration.

#### 2.3.3. Cloud mask

The CALIPSO mission and in particular the Cloud-Aerosol Lidar with Orthogonal Polarization (CALIOP) provides a unique and independent opportunity to evaluate cloud mask products. Despite its relatively narrow footprint (330m to 5km depending on the altitude of the layer sensed), CALIOP acquires data about 2 minutes after MODIS Aqua, which makes it ideal for cloud mask evaluation and the MODIS Aqua cloud mask can then be used itself as a reference. The current AVHRR cloud mask has been evaluated against MODIS Aqua and the results show that is improved as compared to the CLAVR algorithm [10]. This improved technique utilizes albedo thresholds derived from MODIS Aqua data to mask clouds.

#### 2.3.4. Surface Reflectance accuracy assessment

Accurate estimation of atmospheric parameters, such as water vapor content or aerosol optical thickness, is critical and comprises the main source of error in the surface reflecatance estimation. With the purpose of assessing the performance of the AVHRR surface reflectance product, we compare it with the surface reflectance derived from the top of the atmosphere AVHRR data corrected using field-based atmospheric data. These data were extracted for over 48 AERONET sites distributed across the globe [9].

#### 2.3.5. Direct intercomparison of the surface reflectance products

Inter-comparison of the surface reflectance products from different sensors can be used to evaluate their performance and check their inter-consistency. The MODIS data are accurately calibrated and the surface reflectance product has been validated through the various stage (up to Stage III) defined by the MODIS land validation approach [21]. Thus, the MODIS surface reflectance product can be considered as a good reference to evaluate the AVHRR surface reflectance product. The AVHRR surface reflectance and MODIS Aqua data over the BELMANIP2 (BEnchmark Land Multisite ANalysis and Intercomparison of Products) sites were intercompared, using the directional [22] correction. BELMANIP2 is an updated version of BELMANIP1 [23] that aims at providing a representative set of relatively flat and homogenous sites sampling the variability of land surface type and state over the globe. The original BELMANIP2 dataset included 445 sites (Figure 7).

#### 2.3.6. Agriculture application

As a demonstration of the utility of the LCDR, we apply the methods developed by [1] and [2] to test the performance of the AVHRR data to monitor wheat yield. These methods are based on the assumption that the yield is positively and linearly correlated to the seasonal maximum NDVI (adjusted for background noise) at the administrative unit (AU, county or oblast) level and to the purity of the wheat signal (percentage of wheat within the pixel). [1] developed a regression model that was calibrated and applied at the state level in Kansas using MODIS data and proved to be directly applicable at the national level in Ukraine. Looking for an improvement in the timeliness of the yield forecast, [2] enhanced the [1] method by including growing degree day (GDD) information. With this method a reliable forecast can be made between 30 days to 45 days prior to the peak NDVI (i.e. 60 to 75 days prior to harvest), while keeping an accuracy of 10% in the yield forecast. Note that this method provides the same yield results than [1] when the yield forecast is applied during the date of the NDVI peak. In this work, we evaluate the yield model’s applicability to AVHRR.

## 3. Results

### 3.1. Geolocation

**Figure 1.**
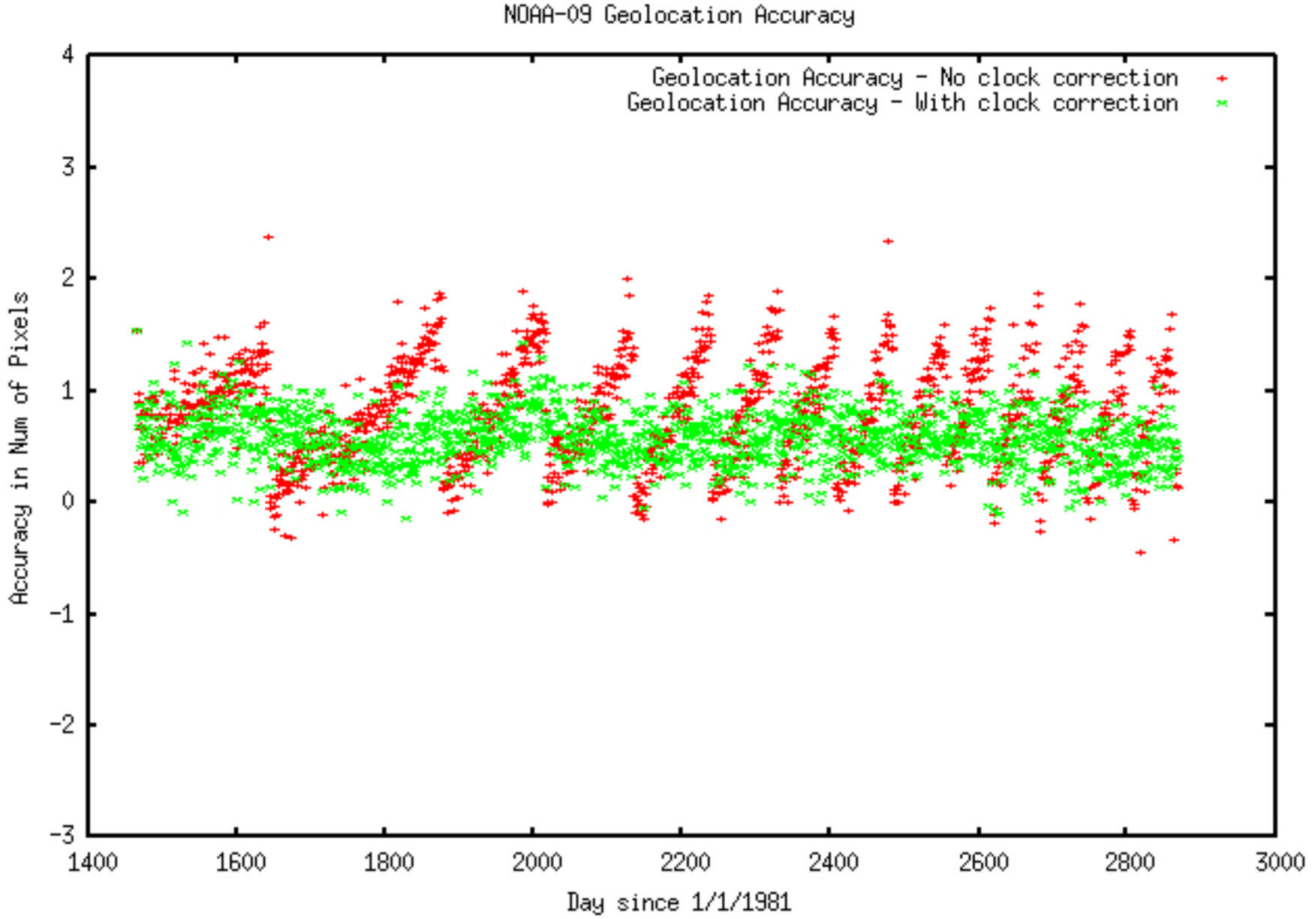
Accuracy assessment of the geolocation of AVHRR products using the coastal chips database (in fraction of pixels). Green is with clock correction, red is without clock correction.

Geolocation is an important prerequisite to ensure consistency in the land time-series of observations [11]. A number of physical effects such as clouds, atmospheric contamination and surface anisotropy require compositing multiple daily orbits into a single data set [12,13]. Achieving a high-level accuracy of relative geolocation is a critical step for each orbit [14]. Therefore, major efforts are made in geometric correction and the assessment of geolocation accuracy. The accuracy of this correction was assessed by using the coastal chips database as a reference. When the on-board clock was reset, a discontinuity in the accuracy is introduced (Figure 1, red dots). The clock correction approach developed by [15] improves significantly the geolocation accuracy (Figure 1, green dots).

### 3.2. Calibration monitoring

**Figure 2:**
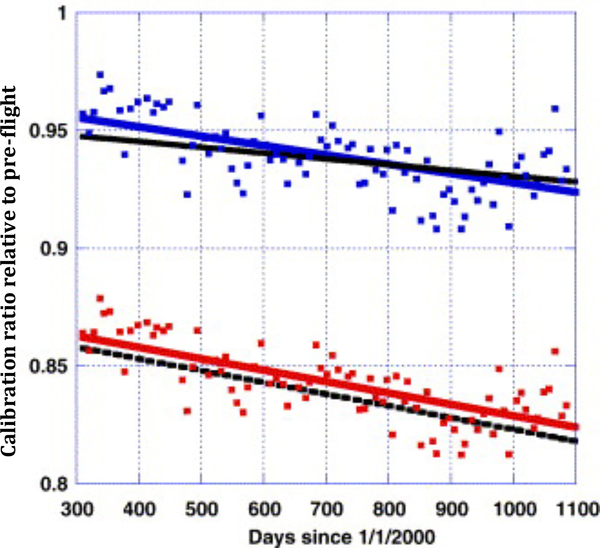
Comparison of the NOAA16-AVHRR/MODIS Terra cross calibration over desert sites for band 1 (black solid line) and band 2 (black interrupted line), with the trends obtained using the Ocean and Clouds method [4] for band 1 (blue line and square) and band 2 (red line and square) (from [5]).

Accurate radiometric calibration is a prerequisite to creating a science-quality time-series of BRDF corrected surface reflectance and consequently, higher order downstream products. Calibration errors can propagate directly into the surface reflectance and create artificial variations that can be misinterpreted as trends, especially if these variations are due to a slow decay in the calibration mechanism. Vicarious calibration provides an additional source of calibration information, to verify and evaluate on-board calibration. As mentioned in the methods section, we will use the approach of [5] for cross calibration of AVHRR with MODIS to monitor the calibration in the visible to shortwave infrared bands and to provide correction terms as needed (Figure 2). To assess this approach, [5] applied it to transfer the MODIS Terra calibration to the MODIS Aqua instrument. When applied to a stable desert ground site in Niger, the results of this approach agreed to within 1% of the MODIS Aqua on-board solar diffuser [5]. The calibration coefficients used are available from the project website (http://ltdr.nascom.nasa.gov).

### 3.3. Cloud mask

**Figure 3.**
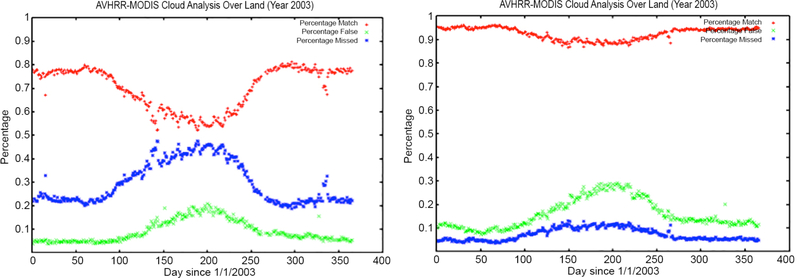
Evaluation of the global performance of the current cloud mask for NOAA16-AVHRR versus MODIS Aqua cloud mask. Results reported as percentage. Left side is the CLAVR algorithm [10]. Right is the current LCDR improved cloud mask. MODIS Aqua cloud mask is used as truth in this comparison. Red symbols (Match) show the percentage of agreement between AVHRR and MODIS, Green symbols (False) show the percentage of cases where AVHRR erroneously detects clouds, Blue symbols (Missed) show the percentage of cases where AVHRR missed clouds.

**Figure 4.**
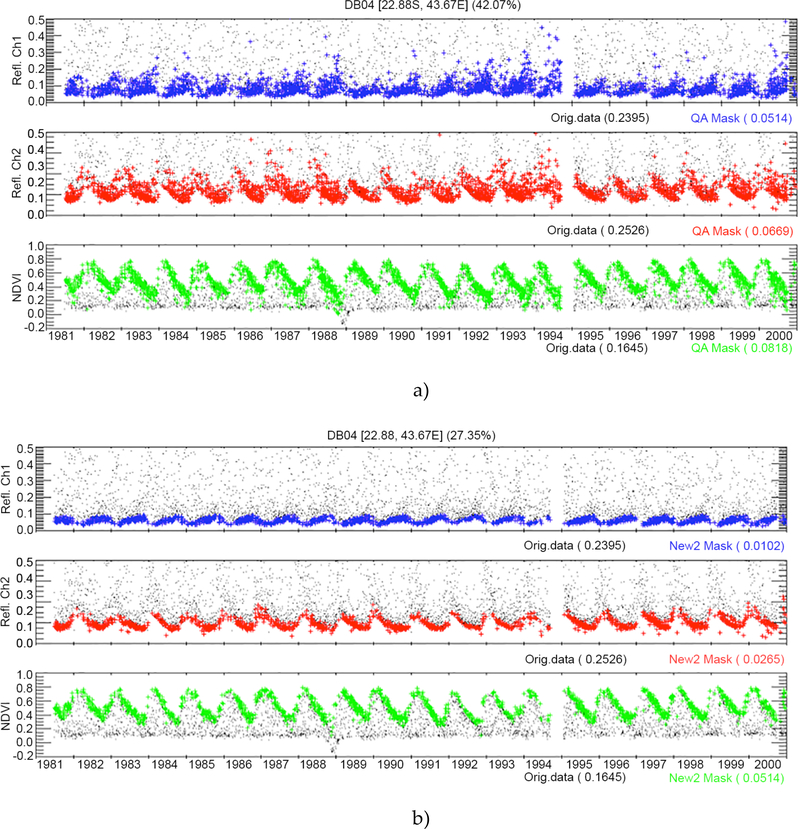
AVHRR time-series of channel 1 (blue) and channel 2 (red) surface reflectance and the NDVI (green) using a) CLAVR or b) LCDR cloud masks for a deciduous broadleaf site in Madagascar. Black symbols are clouds. The standard deviation of the unfiltered data of the time series (Orig.data) and of the cloud filtered time series (QA Mask for CLAVR, New2 Mask for LCDR cloud mask) are also provided for each of the bands and the NDVI. The percentage of clear data is also provided for each cloud mask at the top of the figure.

While the validation of surface reflectance is facilitated by AERONET data, the validation of the cloud mask remains a significant challenge. To verify the improvement in the cloud mask, we have undertaken an inter-comparison between the AVHRR cloud mask with the MODIS Aqua cloud mask for near-coincident (in time) observations. Figure 3 shows the evaluation of the improved AVHRR cloud mask, where the agreement with MODIS Aqua is higher than 90% compared to an average 60% agreement for the CLAVR cloud mask. Figure 4 shows the time-series evolution of the surface reflectance of channel 1 (blue) and channel 2 (red) as well as the NDVI (green) over one BELMANIP2 site (see section 2.3.4 for a description of the BELMANIP2 sites) located in Madagascar, using the CLAVR cloud mask (Figure 4a) and the LCDR cloud mask (Figure 4b). These plots show a strong reduction of noise when using the LCDR cloud mask in channel 1 (from 0.05 to 0.01), channel 2 (from 0.07 to 0.03) and the NDVI (from 0.08 to 0.05).

### 3.4. Surface Reflectance accuracy assessment

**Figures 5.**
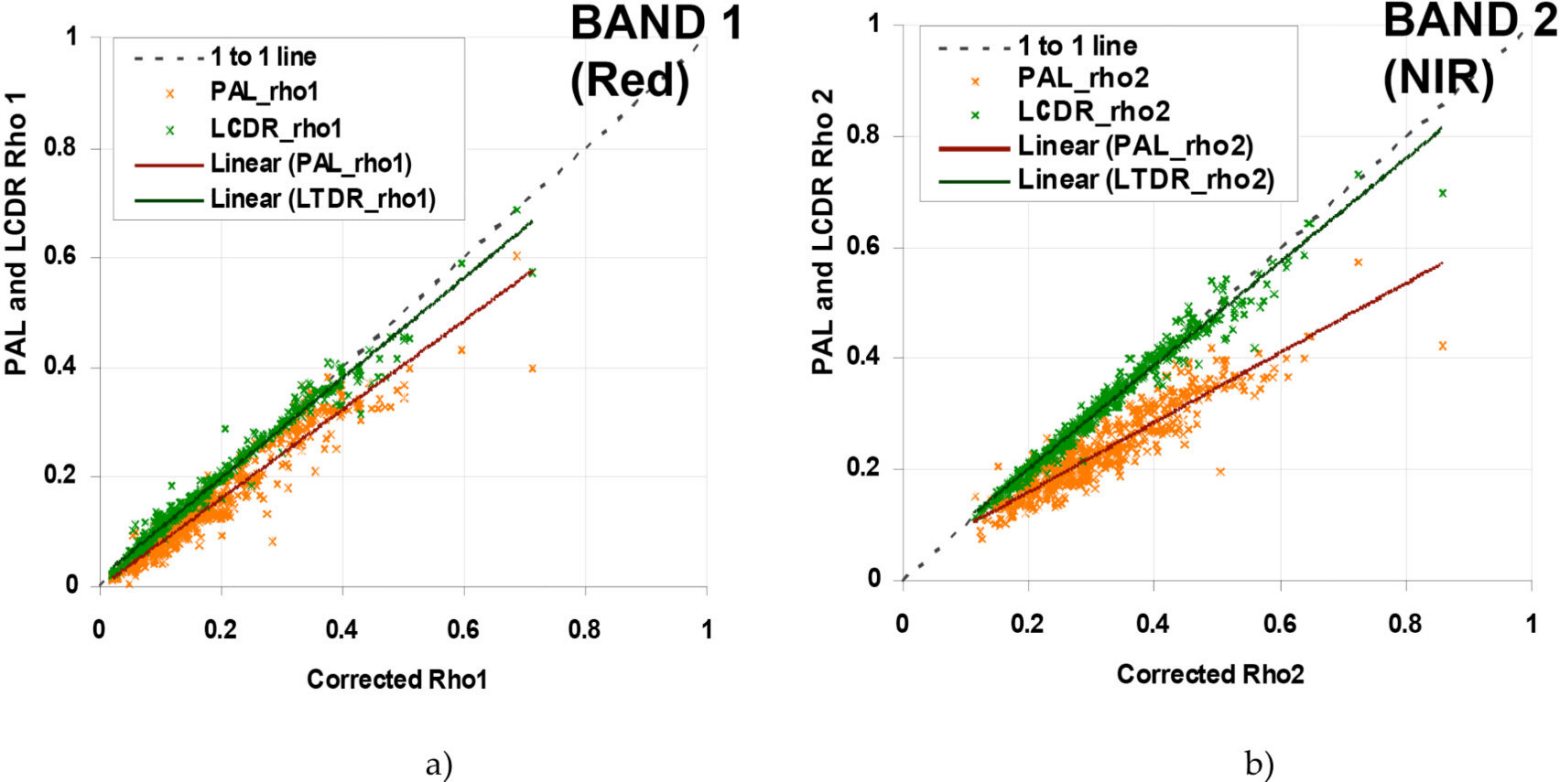
Comparison of current AVHHR Surface Reflectance (LCDR) and PAL data for channel 1 (a) and channel 2 (b) at 48 AERONET sites for 1999 (from [9]). The x-axis shows the surface reflectance values determined from the 6S code supplied with atmospheric parameters from an AERONET sunphotometer, while the y-axis shows the surface reflectances retrieved from the AVHRR data using current LCDR and PAL algorithms.

We have analyzed a comprehensive estimate of the performance of the AVHRR Surface Reflectance for 1999 over the AERONET sites [12]. The performance was evaluated along with Pathfinder AVHRR Land (PAL) daily products [16] over 48 sites distributed across the globe [9]. Atmospheric data from AERONET sunphotometers at each site [17] were used as input to the 6S radiative transfer model [18] to atmospherically correct the top of the atmosphere AVHRR data to determine surface reflectance values for channels 1 and 2. Figure 5a shows that the AVHHR data for channel 1 follow the one-to-one line very closely. Similarly, Figure 5b shows the AVHRR results for channel 2, with good correlation for surface reflectance values up to ~0.5, although the PAL data are further from the 1-to-l line.

### 3.5. Direct intercomparison of the surface reflectance products

**Figures 6.**
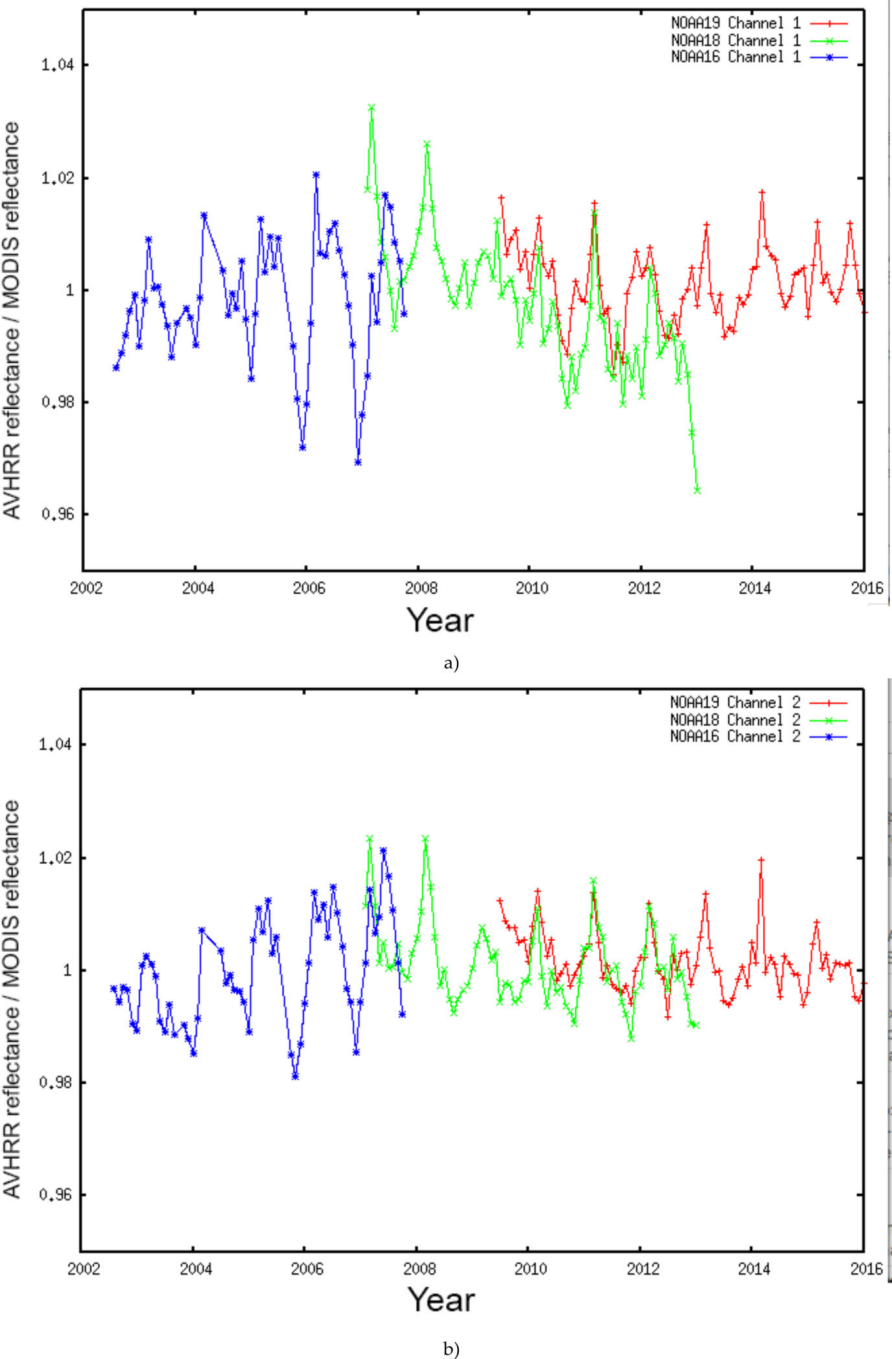
Cross comparison between AVHRR N16, N18 and N19 and MODIS Terra ratios for the BELMANIP2 sites for the red band (a) and the near infrared band (b).

Figure 6 shows the cross-comparison of AVHHR data with MODIS over the BELMANIP2 sites. The monthly averaged ratios of the observed (AVHRR data) and the predicted reflectance (MODIS Aqua corrected reflectance at AVHRR spectral and directional conditions) for AVHRR channel 1 (Figure 6-top) and channel 2 (Figure 6-bottom) are plotted as a function of time [5]. The plots show a consistent evolution of the ratios for the different sensors (NOAA16, NOAA18 and NOAA19) and for the two channels with values close to one. It should be noted, that at the beginning of each mission there are discrepancies between sensors [19] (beginning of the NOAA18 record with NOAA16 and beginning of the NOAA19 record with NOAA18), this is expected during the outgassing period where both the thermal bands are not stable and the calibration in the red and near infrared is evolving quickly.

### 3.6. Derived LAI/FAPAR products

**Figure 7.**
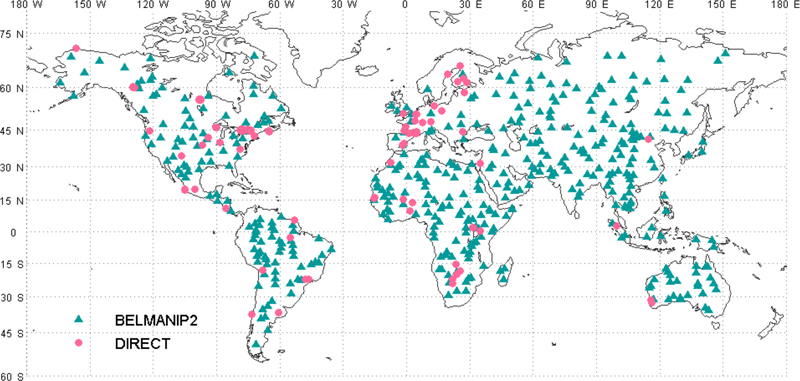
BELMANIP-2 and DIRECT network sites location (http://calvalportal.ceos.org/web/olive/site-description).

**Figure 8.**
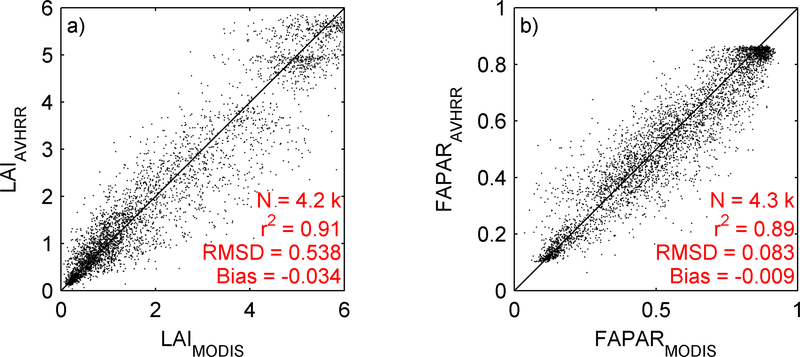
Comparison of MODIS and AVHRR LAI (a) and FAPAR (b) during 2001 to 2007. Data were extracted over DIRECT sites not used during the training process.

Using AVHRR surface reflectance, a LAI/FAPAR product (AVH15C1 product) was derived [20]. The algorithm relies on Artificial Neural Networks (ANN) trained using MODIS LAI/FAPAR products and AVHRR surface reflectance products, acquired over BELMANIP-2 sites from 2001 to 2007. A full description of the algorithm and its evaluation process is given in [19]. Using different sites than the ones used for training (DIRECT network, [22], Figure 7), Figure 8 shows that the MODIS and AVHRR LAI/FAPAR are well correlated (r^2^~0.9). However, a clear saturation effect is observed with high FAPAR (>0.8) values. This saturation affects mainly deciduous forest, associated with a complex 3D canopy [21].

### 3.7. Agriculture application

With the purpose of evaluating the applicability of the yield models to AVHRR data, we validate the methods taking advantage of the AVHRR LTDR historical data from 1982 to 2014.

**Figure 9.**
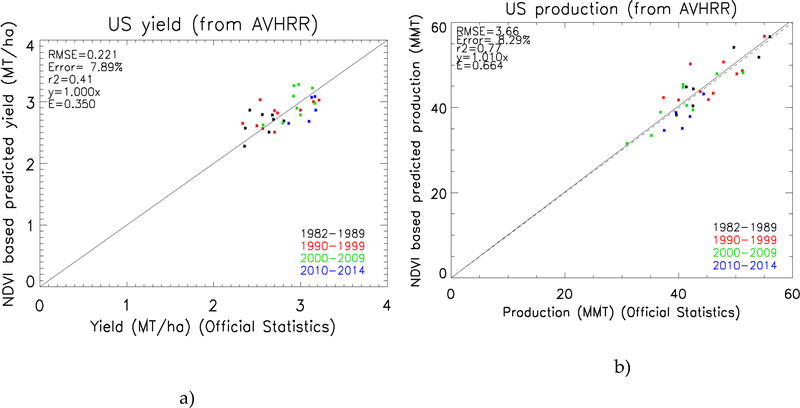
National winter wheat predicted yield (a) and production (b) in the U.S., applying the [1] ‘original’ method to AVHRR data plotted against USDA reported statistics (https://quickstats.nass.usda.gov).

Figure 9 shows the validation of the method using the AVHRR LCDR data from 1982 to 2014. Note that we removed from the analysis the year 2007 that was identified as a problem in [1], when a late frost damaged most of the wheat crops in Kansas and Oklahoma. Comparing the statistics of these figures with the statistics presented in [2], where the model is applied using MODIS data from 2001 to 2012, adding more years to the analysis and using Version 4 AVHRR surface reflectance data barely affects the error, keeping it at around 7%. These results confirm the good performance of the method, providing good results during the extreme years in terms of production. The statistics also display the Nash-Sutcliffe model efficiency coefficient (E) proposed by [23]. It is defined as one minus the sum of the absolute squared differences between the predicted (P) and observed (O) values, normalized by the variance of the observed values during the period under investigation.

$$E=1-\frac{\sum_{i=1}^{n}{\left(O_{i}-P_{i}\right)}^{2}}{\sum_{i=1}^{n}{\left(O_{i}-{\bar{O}}_{i}\right)}^{2}}$$

The range of E lies between 1.0 (perfect fit) and −∞. An efficiency of lower than zero indicates that the mean value of the observed time series would have been a better predictor than the model. Both the yield and the production show E values greater than zero.

**Figure 10.**
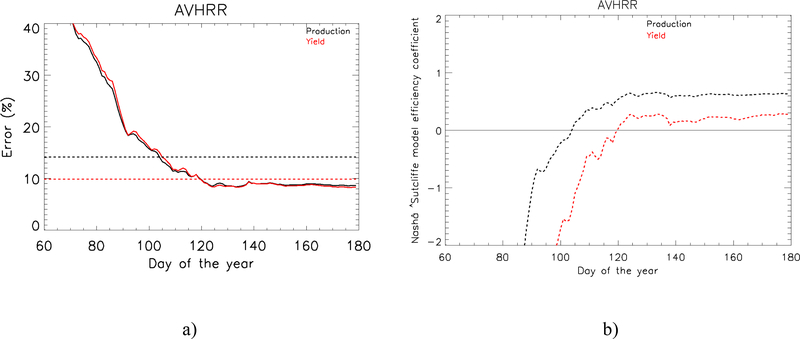
a) Percentage error evolution when forecasting the winter wheat production (black) and yield (red) with historical AVHRR data. The dashed line represents the error committed when considering a constant production (black) or yield (red) and equal to the average through the time series. b) Nash–Sutcliffe model efficiency coefficient evolution depending on the day of the year of the forecast.

Figure 10 shows the error evolution of the yield and the production when applying the [2] method depending on the day of the forecast. Comparing this plot to the results published in [2] that was just based on the MODIS-era time-series, shows that the inclusion of more days in the analysis provides more stability in the error evolution. The plot also shows a horizontal line that represents the error if we assume the yield/production equal to the time series average. In order to study the feasibility of the model compared to assuming the average yield/production, Figure 10 displays the evolution of the E coefficient. The yield forecast shows E positive values up from DOY 120 (April 30^th^), while for the production which is corrected by the official statistics of area, the E coefficient is positive from DOY 100 (April 10^th^).

**Figure 11.**
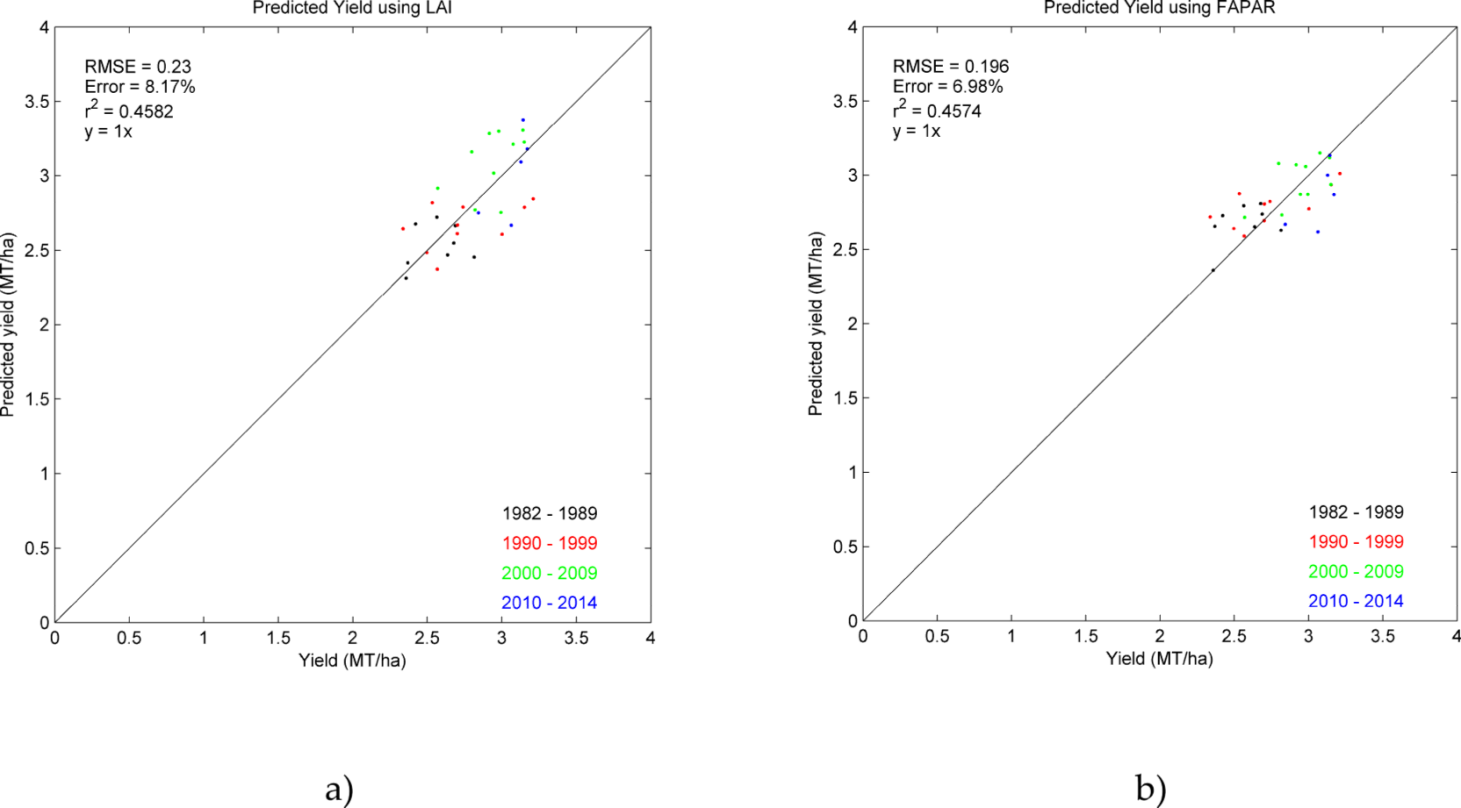
National winter wheat predicted yield in the U.S. applying [1] method to LAI (a) and FAPAR (b) AVHRR data.

We also used the AVH15C1 LAI and FAPAR products with the [1] method. Figure 11 shows the yield validation with the official statistics. Comparing these results with the NDVI (Figure 11a), they show similar errors (8.07% NDVI, 8.17% LAI and 6.98% FAPAR) and similar correlation coefficients (0.38 NDVI, 0.46 LAI and 0.46 FAPAR). Thus, we can conclude that the three different parameters (NDVI, LAI and FAPAR) provide equivalent results.

## 4. Discussion

In this work we present the improvements and assess the AVHRR BRDF corrected surface reflectance/NDVI Version 4 product. Besides the geolocation and cloud mask evaluations, the assessment is done through four different exercises: first, we compare the product with the surface reflectance derived using AERONET atmospheric data (section 3.4); second, we intercompare the AVHRR with the MODIS surface reflectance product; third, we evaluate the LAI and FAPAR downstream products; and fourth, we apply a method to the AVHRR historical surface reflectance dataset to estimate the wheat production in the U.S.

The inter-comparison of MODIS and AVHRR surface reflectance products show ratios close to one, which means that both time-series are consistent. However, the ratio still shows some noise (maximum of 2% variation). The reasons for such errors could be associated with errors in the water vapor correction, an error residual of the BRDF correction or even a systematic variation of the calibration during the year. All these possible explanations will be further explored in our future work.

Regarding the yield model, the method developed for MODIS data was evaluated with the longer AVHRR historical record, which contains greater inter-annual variability in surface conditions (generally winter wheat yields with lower values: see x-axis data variability of Figure 11a). Additionally, the method was applied satisfactorily to AVHRR using the same calibration coefficients as for MODIS and producing equivalent statistics, showing the comparability and consistency of the MODIS and AVHRR surface reflectance products for this application.

## 5. Conclusions

This paper evaluated the AVHRR BRDF corrected surface reflectance/NDVI Version 4 product. We reviewed the various efforts developed to improve its accuracy, from the geolocation correction and the cloud mask improvement to the calibration monitoring. Additionally, we evaluated the performance of the product, first using AERONET data and also by inter-comparison with the MODIS surface reflectance, an already validated and established product. The results presented show good performance of the AVHRR product and consistency with MODIS. We also demonstrate the usefulness and assess the performance of the product by its application to agricultural monitoring. This agricultural application demonstrates the utility of the LCDR to test the robustness of the yield forecast methods.

We are still working on the improvement of the product based on a better estimation of the atmospheric constituents: the aerosols and water vapor content. Future work will also include the development of a more systematic, robust and statistically significant evaluation of the product.
